# Full-Spectrum Hyperspectral Modeling of Leaf Dry Matter Content Using a Stacked Ensemble Framework

**DOI:** 10.3390/s26051665

**Published:** 2026-03-06

**Authors:** Reinis Alksnis, Ina Alsina, Mara Duma, Laila Dubova, Uldis Gross, Tetiana Harbovska

**Affiliations:** 1 Faculty of Engineering and Information Technology, Latvia University of Life Sciences and Technologies, LV-3001 Jelgava, Latvia; uldis.gross@lbtu.lv; 2 Faculty of Agriculture and Food Technologies, Latvia University of Life Sciences and Technologies, LV-3001 Jelgava, Latvia; ina.alsina@lbtu.lv (I.A.); mara.duma@lbtu.lv (M.D.); laila.dubova@lbtu.lv (L.D.); tetiana.harbovska@lbtu.lv (T.H.)

**Keywords:** dry matter, stacking models, light reflectance spectra, crops, water indices

## Abstract

The objective of this study was to assess the predictability of leaf dry matter content across a diverse range of plant species using hyperspectral reflectance data. The dataset encompassed leaves from multiple crops, including potatoes, beans, wheat, maize, peas, tomatoes, basil, and cucumbers, collected under varying growth conditions, cultivation systems, seasonal contexts, and developmental stages. As an initial benchmark, commonly used narrow-band spectral indices and their combinations were evaluated, but they exhibited limited predictive performance for dry matter content. Consequently, several full-spectrum machine learning models were trained and compared to assess their individual predictive ability. Given their complementary strengths, these models were integrated into a stacked ensemble framework to enhance overall accuracy. The resulting ensemble, combining the outputs of multiple base learners through a meta-learner, achieved a coefficient of determination of $R^{2}=0.896$ on an independent test set, outperforming all individual models. The findings highlight the potential of a multi-model stacking approach to improve the accuracy and robustness of leaf biochemical property estimation from hyperspectral data.

## 1. Introduction

Dry matter in plants is produced primarily through photosynthesis. During this process, leaves convert light energy into chemical energy and synthesize carbohydrates from carbon dioxide and water. These assimilates contribute to the formation of structural and storage components of the plant and serve as key indicators of plant growth, water-use efficiency, and potential yield [1,2]. Formed compounds are transported to leaves, stems, roots, and reproductive structures, leading to increased biomass and dry matter accumulation over time. The total amount of dry matter is influenced by different factors such as light intensity, water availability, and nutrient supply as well as leaf area development and pigment (especially chlorophyll) content [3].

Dry matter content and water content in plants are closely related, as they together form the total biomass: water is essential for photosynthesis, nutrient transport and cell growth, while dry matter characterizes the organic and inorganic compounds that are produced as a result of photosynthesis [1]. An optimal amount of water allows plants to synthesize carbohydrates and other compounds, increasing the accumulation of dry matter, while water deficit can limit photosynthesis and reduce the formation of dry matter [3].

The ratio of dry matter to water content is an important indicator of both plant growth and plant stress. Water content and dry matter accumulation are affected by factors such as soil moisture, temperature, plant species, plant age, etc. Scientists have found that plants affected by drought stress sometimes have a higher percentage of dry matter, despite a lower total biomass [4].

Gravimetric analysis is a classic chemical method used to determine dry matter content by measuring the weight change between fresh and dried leaves, but it is a destructive, time-consuming and energy-consuming method. Non-destructive methods are often considered superior to biochemical methods because these methods offer advantages in terms of sample preservation, long-term monitoring, minimal impact and cost-effectiveness [5,6].

Reflectance spectroscopy is widely used in plant stress studies [7,8], as the spectra obtained can characterize the health status of plants, stress level, productivity and even the content of certain components, such as chlorophyll or flavonoids. For example, reflectance spectra containing information about pigments are found in the visible (VIS) or infrared region, while changes in leaf structure are characterized by spectra in the near infrared (NIR) region [9]. Readings in the near-to-mid-infrared region are recommended to obtain information about dry matter/water content [9]. Reflectance spectroscopy and hyperspectral reflectance measurements have been widely used to estimate water content in plants [10] and are valuable tools for assessing the water content of a variety of materials, including soils, plants, and water bodies [11].

Recent studies on dry matter estimation in plants and crops have highlighted the need to develop non-destructive and high-throughput methods for biomass and dry matter estimation, replacing traditional destructive detection methods. Studies using unmanned aerial vehicles (UAVs) equipped with RGB and hyperspectral sensors combined with machine learning have shown promising accuracy in estimating dry matter and forage yield in grasslands and crops, offering practical tools for precision agriculture [12]. Remote sensing approaches that combine vegetation indices with phenological variables have been successfully applied to estimate biomass in cereals, reflecting the integration of spectral data and crop growth models [13]. Studies have also shown that hyperspectral spectroscopy combined with artificial intelligence can effectively model dry matter and nutrient content, highlighting wavelength regions that are sensitive to biomass components [4]. There are studies that confirm that dry matter content modeling can serve as an estimator of total biomass production, aiding in ecological and agricultural monitoring. Collectively, these advances illustrate how combining remote sensing, machine learning, and phenotypic data improves the accuracy, speed, and applicability of dry matter estimation in diverse plant systems [14].

Because hyperspectral reflectance data are inherently high-dimensional and exhibit strong collinearity across neighboring wavelengths, dry matter estimation is typically formulated as a multivariate regression problem. Classical chemometric approaches such as principal component regression (PCR) and partial least squares regression (PLSR) have long been used to relate spectral features to biochemical and structural plant traits, while more recent studies increasingly rely on machine learning algorithms including random forests, gradient boosting, kernel methods, and neural networks. These models are capable of capturing nonlinear responses and complex feature interactions distributed across the spectrum and have repeatedly demonstrated improved predictive performance compared with single-index approaches in vegetation spectroscopy and plant-trait retrieval studies [15,16,17]. However, comparative studies also show that no single algorithm consistently performs best across datasets, species groups, and measurement conditions, suggesting that different learners capture complementary aspects of the spectral signal.

One principled way to exploit such model complementarity is ensemble learning, where predictions from multiple base models are combined into a single meta-model. Among ensemble strategies, stacked generalization, introduced by Wolpert [18], has been shown to reduce generalization error by learning how to optimally combine heterogeneous base learners through a second-level model. Different types of base learners can be used in the first level, among those all the models mentioned above. Generally, in this first level, it is useful to include models that complement each other. For instance, linear models capturing general linear associations should be complemented with nonlinear models to capture higher, more nuanced nonlinearities. Stacking ensemble learning has gained popularity in recent years with applications, among others, in agriculture and remote sensing. Fu et al. (2019) [19] combined partial least squares regression (PLSR), random forests (RF) and Gaussian process regression (GPR) to build a model for predicting photosynthetic capacity from hyperspectral reflectance spectra of tobacco leaves. Yoosefzadeh-Najafabadi et al. (2021) [20] used a stacking model with support vector regression, random forests and neural networks as base learners to predict soybean yield from hyperspectral reflectance and associated physiological traits. Chen et al. (2022) [21] proposed a stacked ensemble extreme learning model by integrating kernel-based and tree-based models to predict nitrogen, phosphorus and potassium concentrations in apple tree leaves using canopy hyperspectral data. Dumancas and Adrianto (2022) [22] stacked partial least squares, support vector machines and artificial neural networks to predict biomass composition from near-infrared spectroscopy data. Fei et al. (2023) [23] introduced a feature splitting regression technique, conceptually related to stacking, for predicting plant biochemical traits from high-dimensional reflectance data. Sharma et al. (2024) [24] extended the stacking concept to integrate convolutional neural networks with ensemble regression models for predicting grapevine physiological parameters from hyperspectral data. Abukmeil et al. (2026) [25] developed a multivariate stacked regression model for predicting correlated macro- and micronutrient concentrations in potato plants using visible and near-infrared reflectance. Li et al. (2025) [26] proposed a stacking ensemble combined with a one-dimensional convolutional neural network model for chlorophyll content prediction in Stellera chamaejasme leaves. These are just a few instances of the vast literature from recent years establishing that stacked ensemble learning is an effective strategy for hyperspectral-reflectance-based predictions of various plant biochemical traits. Thus, our strategy is also to combine linear models (PCR, PLSR, Enet) and nonlinear models (RF, XGBoost, LightGBM, GPR) with the idea that stacking would capture complementary variance structures and thus enhance predictions accuracy, improving the one obtained by the base learners given above.

The aim of this study is to evaluate the predictive limits of hyperspectral data for estimating leaf dry matter by testing a broad range of regression algorithms applied to spectral principal components. Specifically, we investigate whether combining complementary learners in stacked ensembles can yield more robust and accurate predictions than individual models. To achieve this, we integrate both classical and modern regression approaches, ultimately constructing a stacked ensemble framework capable of capturing diverse spectral–dry-matter relationships across multiple crop species.

## 2. Materials and Methods

### 2.1. Plant Cultivation and Sampling

The experiments were established in 2024 and 2025 at the experimental field (EF), vegetation polygon (VP), greenhouses (GHs), and growth chamber (GC) of the Institute of Soil and Plant Sciences of the Latvia University of Life Sciences and Technologies. Both field experiments (FEs) and experiments in vegetation containers (VCs) were conducted. Various types of experiments (Table 1) were set up:

- Fertilizers (F): mineral (FM), organic (FO), and organic granulated (FG) fertilizers were balanced according to the nitrogen dose, F0—no additional fertilizer, F1—N dose 50 g m^−2^, F2—100 g m^−2^.
- Plant irrigation regimes (W): WS sprinkler irrigation, WN without additional irrigation, W1 irrigated each second day, W2 irrigated once a week.
- Different light conditions (L): L1 blue and red light together made up more than 90% of visible light, L2 blue and red light together made 80% of visible light, L3 blue and red light together made 50% of visible light.
- Microbial preparation (M) *Priestia megaterium* was added to substratum (M1), without microbial preparation (M0).
- Soil management methods (S) reduced tillage (without soil conversion) (S0) and conventional tillage (ploughing) (S1).

### 2.2. Reflectance Spectra and Dry Matter Analysis

Reflectance spectra of plant leaves were obtained using an RS-3500 portable spectroradiometer (Spectral Evolution Inc., Haverhill, MA, USA). Reflectance was measured with 1 nm resolution in the 350–2500 nm range. The leaf was detached, and reflectance in three replicates were immediately measured directly from the leaf surface. The same leaves were immediately weighed and placed in a thermostat for dry matter analysis. Leaves were dried at 60 °C for 24–48 h until a constant weight was achieved. Dry matter (DM) content of leaves was measured by weighing the leaves before ($m_{fresh}$) and after drying ($m_{dry}$) and calculated as follows (1):

(1) $$\mathrm{DM}\left(\%\right)=\frac{m_{dry}}{m_{fresh}}\times 100\%$$

### 2.3. Data

**Figure 1:**
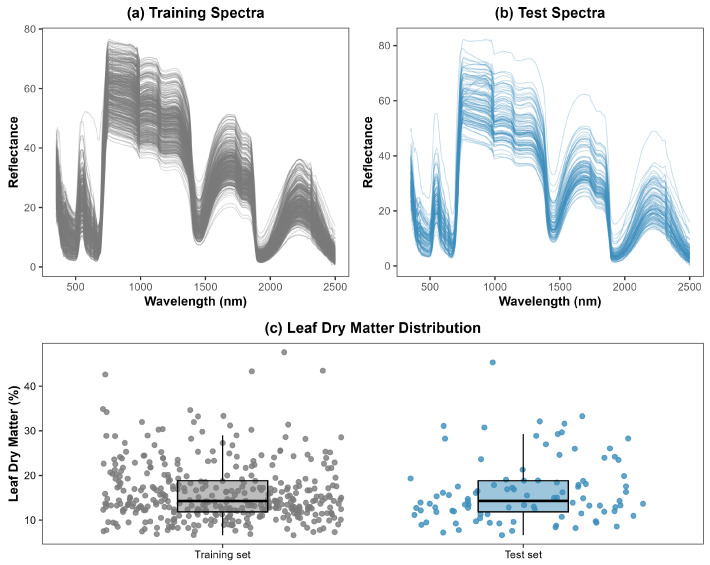
Comparison of spectral reflectance and dry matter distributions for training and test datasets. (**a**) Reflectance spectra (350–2500 nm) for training samples (gray) showing the typical VIS–NIR–SWIR leaf reflectance profile, with high variability in the near-infrared region due to internal leaf structure. (**b**) Reflectance spectra for test samples (blue), displaying a similar overall spectral shape, indicating consistent spectral calibration and sampling between datasets. (**c**) Distribution of measured leaf dry matter (%) for the training (gray) and test (blue) sets, shown as boxplots with overlaid jittered points. The similar ranges and medians between datasets confirm that the training and test partitions are spectrally and biochemically comparable, supporting robust model generalization.

The total dataset comprised 482 leaf samples, each characterized by spectral reflectance values measured from 350 to 2500 nm and the corresponding leaf dry matter content (%). The dataset was randomly partitioned into training (80%;n=384) and testing (20%;n=97) subsets to enable model calibration and independent performance evaluation (see Figure 1). For reproducibility, the random number generator was initialized using set.seed(2025) in *R* prior to splitting the dataset, ensuring that the same training and testing subsets could be obtained in repeated analyses. Samples were randomly partitioned into training and test sets to evaluate generalization within the pooled dataset. Metadata identifying crop species, year, and growth conditions were not retained at the individual-sample level in the modeling table; therefore, we could not perform species or condition-blocked validation. As a result, the random split may partially share species- or batch-related structure between training and test sets; thus, the reported metrics should not be interpreted as strict cross-species or cross-experiment generalization. The reported performance should be interpreted as within-protocol generalization for spectra acquired using the same instrument and measurement setup.

To ensure comparability between training and testing subsets, the distributional properties of the dry matter content were statistically examined (see Table 2). The Shapiro–Wilk test was first applied to assess normality. The results indicated significant deviations from normality in both subsets (training: W = 0.8915, *p* < 0.001; testing: W = 0.8877, *p* < 0.001) suggesting a non-normal distribution of dry matter values. Visual inspection of histograms revealed positive asymmetry. Next, the Fligner–Killeen test was used to evaluate the homogeneity of variances between training and testing subsets. The test ($\chi^{2}\left(1\right)=0.789$, *p* = 0.374) indicated no significant differences in variances between the two subsets. Finally, a Mann–Whitney U test (Wilcoxon rank-sum test) was applied to compare the central tendency of dry matter between the subsets. The test showed no statistically significant difference between the groups (W = 18,460, *p* = 0.894), confirming that the training and testing samples were drawn from comparable distributions. Overall, these results demonstrate that the training and testing subsets did not significantly differ in terms of distribution of variance.

### 2.4. Preprocessing and Feature Extraction

Reflectance spectra were recorded from 350 to 2500 nm at 1 nm resolution, resulting in 2151 spectral predictors per sample. Prior to modeling, data were visually inspected for outliers and missing values. Spectra were visually inspected for measurement artifacts, including abnormal reflectance magnitude, discontinuities, or physically implausible spectral shapes. One spectrum exhibited a clear anomaly characterized by an irregular reflectance pattern inconsistent with typical leaf optical properties, likely due to improper sensor–leaf contact during acquisition. This observation was therefore removed prior to analysis. No additional automated multivariate outlier detection procedures (e.g., Mahalanobis distance or Cook’s distance) were applied, as no further spectra displayed extreme leverage or atypical structure. All reflectance variables were mean-centered and standardized to unit variance to ensure equal contribution of each wavelength. To evaluate whether noise reduction could improve predictive performance, spectra were optionally smoothed using a Savitzky–Golay (SG) filter (polynomial order 2; window sizes 7, 9, 11, and 13 bands). Although SG filtering is commonly used to suppress high-frequency sensor noise while preserving absorption features, it did not improve cross-validated performance for any of the evaluated models and in most cases slightly degraded accuracy. This is likely because the raw reflectance spectra, acquired under controlled laboratory conditions, already exhibited a high signal-to-noise ratio, and smoothing may have attenuated subtle SWIR features associated with dry-matter-related structural absorption.

Although plants were cultivated under diverse environmental and treatment conditions (Table 1), all spectral measurements were acquired under controlled laboratory conditions using a fixed leaf-clip geometry and identical instrumentation. Consequently, no atmospheric or scatter corrections (e.g., SNV or MSC) were applied; standardization and PCA-based modeling were sufficient to control for scale differences and collinearity.

### 2.5. Spectral Indices and SWIR Feature Benchmarking

Before fitting machine learning models, we first evaluated whether dry matter content could be predicted using simple spectral indices derived from known water-sensitive wavelength regions. This step also allowed us to set a benchmark for more complex prediction methods. We examined a set of commonly used reflectance indices and SWIR-based structural proxies (Table 3).

The evaluated indices encompassed a broad range of spectral contrasts sensitive to leaf water status, biochemical composition, and structural properties (Table 3). These included water-related indices targeting absorption features near 970 nm and in the shortwave infrared (e.g., NWI, NDWI, NDII, NDWI-1640, NDWI-Hyperion), lignin- and cellulose-sensitive indices such as the Normalized Difference Lignin Index (NDLI) and the Cellulose Absorption Index (CAI), as well as several stress-oriented ratio indices derived from visible and SWIR bands (e.g., SWSI and DWSI families). In addition, two indices explicitly designed to capture dry matter variation were included, the Normalized Dry Matter Index (NDMI) and the Dry Matter Content Index (DMCI), both of which exploiting reflectance differences in the SWIR region associated with changes in leaf structural and biochemical constituents. Index values were computed separately for the training and test samples. Regression coefficients were estimated exclusively on the training set and subsequently applied to the independent test set to evaluate predictive performance.

### 2.6. Base Learners

Machine learning regression models can be broadly grouped into several categories, each with distinct strengths and weaknesses. Linear models assume a linear relationship between predictors and response, making them interpretable and computationally efficient, but they often struggle with complex, nonlinear patterns. Ensemble methods combine multiple learners to improve prediction accuracy. For instance, bagging approaches, such as random forests, reduce variance by averaging many models trained in parallel on bootstrap samples, while boosting algorithms, such as XGBoost, sequentially correct the errors of previous models, reducing bias but at the risk of overfitting if not tuned carefully. Kernel-based methods (e.g., support vector regression, Gaussian process regression) can capture complex nonlinearities through flexible similarity functions, offering high accuracy but with increased computational cost and less transparency. Neural networks, including deep learning architectures, are highly expressive and can model intricate feature interactions, yet they require larger datasets, careful regularization, and as black-box algorithms are difficult to interpret. In practice, these categories often complement one another: ensembles can capture nonlinearities missed by linear models, kernel methods can achieve similar flexibility without deep architectures, and neural networks can learn directly from raw, high-dimensional data when simpler models reach their limits.

In this study, eight supervised regression approaches were compared for predicting leaf dry matter content from hyperspectral reflectance data. All models were trained on identically preprocessed predictors. Reflectance spectra were first standardized to zero mean and unit variance and subsequently transformed using principal component analysis (PCA). The number of retained components *K* was treated as a tunable hyperparameter, with an upper bound chosen to capture at least 99% of the cumulative spectral variance. Model hyperparameters, including *K*, were optimized using repeated 10-fold cross-validation (with 3 repeats) on the training set. For each method, the configuration with the lowest cross-validated root-mean-square error (RMSE) within one standard error of the minimum was selected.

For each base learner, a broad hyperparameter search space was defined (Table 4). Final model performance was evaluated on a held-out independent test set using RMSE and the coefficient of determination ($R^{2}$). All modeling was conducted in R (v4.4.1) [42] using the tidymodels framework [43] and associated packages.

We should note that deep learning architectures such as one-dimensional convolutional neural networks (1D-CNNs) have been successfully applied in hyperspectral trait prediction [26]. However, such models typically require substantially larger training datasets to achieve stable generalization due to their high parameterization. Given the moderate sample size in the present study (*n* = 481), we prioritized regression approaches with established robustness in limited-sample, high-dimensional settings. Future work will evaluate deep learning models within the stacking framework when larger datasets become available.

We now give a brief description of each of the learners used. Partial Least Squares Regression (PLSR) is a linear method that projects predictors to a low-dimensional latent space maximizing covariance with the response [44]. It is widely used in chemometrics for spectral data due to its robustness to multicollinearity. PLSR is one the most frequently applied algorithm for modeling biological trait as a function of light reflectance spectrum [45]. As a method that focusses on feature space reduction, it is very useful when the number of independent variables is large compared to the number of observations, as in this study with 2151 variables—light reflectance at each nanometer from 350 to 2500. Principal Component Regression (PCR) is a closely related method. It is a two-stage linear method where predictors are first transformed by PCA and then used in a standard linear regression [46]. Unlike PLSR, PCR does not use the response to guide the component extraction. Elastic Net Regression is a linear model with both L1 (lasso) and L2 (ridge) penalties, allowing variable selection and shrinkage simultaneously [47]. Gaussian Process Regression (GPR) is a non-parametric Bayesian method that defines a prior over functions, updated with observed data via a kernel function [48]. GPR can capture complex nonlinear relationships but scales poorly with large datasets. Random Forest Regression (RF) is an ensemble of decision trees trained on bootstrap samples, with random feature selection at each split [49]. RF handles nonlinearities and interactions well and is robust to overfitting. Extreme Gradient Boosting (XGBoost) is a gradient boosting implementation optimized for speed and accuracy [50]. XGBoost builds trees sequentially, with each tree correcting the residuals of its predecessors, and includes strong regularization. Light Gradient Boosting Machine (LightGBM) is another gradient boosting algorithm [51].

## 3. Results

### 3.1. Spectral Index and Feature Benchmarking

To establish a baseline for predictive performance, simple linear regression models were first evaluated using each spectral index as a single predictor of leaf dry matter content. Across the 26 narrow-band and SWIR-derived indices considered (Table 3), predictive performance on the independent test set was generally limited (Table 5). The strongest individual predictive power was obtained for indices specifically designed to capture dry matter and cellulose-related absorption features. In particular, the Normalized Dry Matter Index (NDMI) achieved the lowest error (RMSE = 5.63 percentage points) leading to the highest coefficient of determination ($R^{2}=0.386$), followed by the Dry Matter Content Index (DMCI) with RMSE = 6.13 and $R^{2}=0.271$. Structural and greenness-related indices, such as CAI and NDVI, exhibited weaker but still measurable associations with dry matter content, while several shortwave stress indices (e.g., DWSI-3, DWSI-4, SWSI-2) explained only a modest fraction of variance ($R^{2}<0.12$). In contrast, many commonly used water-related indices, including NDII, NDWI-1640 and MSI, showed negligible predictive power when used in isolation.

Overall, these results suggest that variation in leaf dry matter content cannot be attributed to a single dominant absorption feature or index. Instead, the signal appears to arise from a combination of biochemical composition, structural characteristics, and water-related properties that are expressed across different parts of the spectrum. The modest and highly variable performance of individual indices underscores the limitations of relying on isolated spectral contrasts. This, in turn, points to the need for modeling approaches that can integrate complementary information from multiple spectral features, providing a stronger motivation for multiple regression and full-spectrum machine learning methods.

Because individual spectral indices captured only a small fraction of the variability in leaf dry matter content, we next investigated whether combining all indices within a single regression framework could improve predictive performance. A multiple linear regression including all indices simultaneously, however, gave unstable coefficient estimates due to strong multicollinearity among indices derived from overlapping spectral regions. To address this, we employed elastic net regression, a regularized linear modeling approach that combines the properties of ridge and lasso penalties. Elastic net minimizes a penalized least squares criterion, shrinking regression coefficients toward zero and setting some of them exactly to zero, thereby stabilizing the model in the presence of correlated predictors and performing implicit variable selection. All indices were standardized (zero mean, unit variance) prior to model fitting so that the resulting coefficients were directly comparable in magnitude. The fitted Elastic Net model retained 18 of the 26 indices as predictors

(2) $$\begin{array}{cc} \hat{\mathrm{DM}}=\; & 16.11-4.38\;\mathrm{NDLI}+4.16\;\mathrm{CAI}-2.20\;{\mathrm{NDWI}}_{\mathrm{Hyp}}+2.12\;{\mathrm{DWSI}}_{1}\\ \; & +1.95\;\mathrm{NDII}+1.81\;{\mathrm{DWSI}}_{5}-1.80\;\mathrm{DMCI}+1.78\;\mathrm{NDMI}-1.48\;\mathrm{WI}\\ \; & -1.06\;\mathrm{SRWI}-0.93\;{\mathrm{SWSI}}_{2}-0.47\;\mathrm{NDVI}-0.46\;{\mathrm{DWSI}}_{2}-0.25\;{\mathrm{DWSI}}_{3}\\ \; & +0.23\;\mathrm{NWI}+0.16\;{\mathrm{NWI}}_{970/900}+0.03\;{\mathrm{NDII}}_{2}-0.001\;\mathrm{NDWI}. \end{array}$$

On the independent test set, the Elastic Net model based on spectral indices achieved an RMSE of 4.01 percentage points and an $R^{2}$ of 0.69, representing a substantial improvement over single-index regressions. The fitted coefficients indicated that predictive signal was distributed across multiple biochemical and water-related spectral contrasts rather than being dominated by a single feature. The strongest positive effects were assigned to cellulose- and dry-matter-related SWIR features, particularly the Cellulose Absorption Index (CAI; +4.16), together with positive contributions from NDII (+1.95) and the dry-matter-oriented NDMI (+1.78). In contrast, several indices linked to absorption features in the SWIR and water-sensitive ratios were assigned negative coefficients, including NDLI (−4.38), NDWI-Hyperion (−2.20), DMCI (−1.80), the Water Index (−1.48), SRWI (−1.06), and SWSI2 (−0.93). Overall, the coefficient pattern supports the view that dry matter estimation benefits from combining indices sensitive to structural constituents (cellulose/lignin-related absorption features) with indices capturing hydration-related variation, yielding a markedly stronger predictive baseline than any single index alone.

### 3.2. Single-Model Baselines

Taken together, the index-based analyses indicated that while targeted spectral indices captured some aspects of dry matter variability, they provided an incomplete representation of the underlying signal. Even when combined within a multivariate framework, these hand-crafted features did not fully exploit the richness of the hyperspectral measurements. This naturally motivated the use of more flexible regression approaches that could draw on a much broader portion of the spectral information. Accordingly, we next considered models trained on dimension-reduced representations of the full reflectance spectrum, which allowed complex and potentially nonlinear relationships between spectral features and dry matter content to be learned while mitigating issues related to high dimensionality and strong inter-band collinearity.

To support the ensemble modeling strategy, a range of regression approaches was considered, including latent-variable linear models (PCR, PLSR), regularized regression (Elastic Net), kernel-based learning (Gaussian Process Regression), and tree-based ensemble methods (Random Forest, XGBoost, and LightGBM). The intention was not to compare these methods in isolation but to assemble a diverse set of base learners that brought different strengths to the problem. Models that emphasize a broad, global structure in the spectrum were combined with others that are better suited to capturing localized or nonlinear relationships, increasing the likelihood that complementary information contained in the hyperspectral data could be effectively leveraged through ensemble learning.

The predictive performance of the individual regression models is summarized in Table 6. Among the evaluated approaches, LightGBM achieved the highest accuracy on the independent test set ($R^{2}=0.879$), followed closely by latent-variable linear models, including PCR, PLSR, and Elastic Net, all of which exhibited nearly identical performance. These results suggest that a substantial fraction of the dry matter signal is encoded in broad, approximately linear spectral structures that are effectively captured by dimension-reduction methods. The near-identical test performance of PCR and Elastic Net ($R^{2}=0.871$) was expected under the present modeling setup because both models were trained on the same PCA-transformed predictors. When the predictive signal is concentrated in a limited number of principal components, ordinary least squares on selected components (PCR) and a regularized linear model (Elastic Net) can converge to very similar fitted functions, particularly when cross-validation selects weak regularization. In this setting, regularization provides little additional benefit beyond dimensionality reduction, resulting in comparable generalization performance for the two methods.

Nonlinear models showed more heterogeneous behavior. Random Forest and Gaussian Process Regression provided moderate improvements over index-based approaches but did not outperform the best linear or boosting-based models, while XGBoost exhibited the lowest predictive accuracy among the tested learners. This variability indicates that no single modeling paradigm fully captures the complexity of the hyperspectral–dry-matter relationship. Instead, different algorithms appear to extract complementary information from the same spectral data, motivating their integration within a stacked ensemble framework.

### 3.3. Stacked Ensemble

A stacked ensemble model was constructed to combine the predictive outputs of multiple tuned base learners into a single, integrated regression framework. Candidate models from each regression family were first optimized using cross-validation and subsequently combined at the prediction level using a regularized linear meta-learner. The meta-learner was implemented as a non-negative LASSO regression, which assigns weights to individual base learners while enforcing sparsity to limit redundancy and overfitting. The final ensemble therefore represents a weighted combination of complementary models rather than reliance on a single best-performing approach.

**Figure 2:**
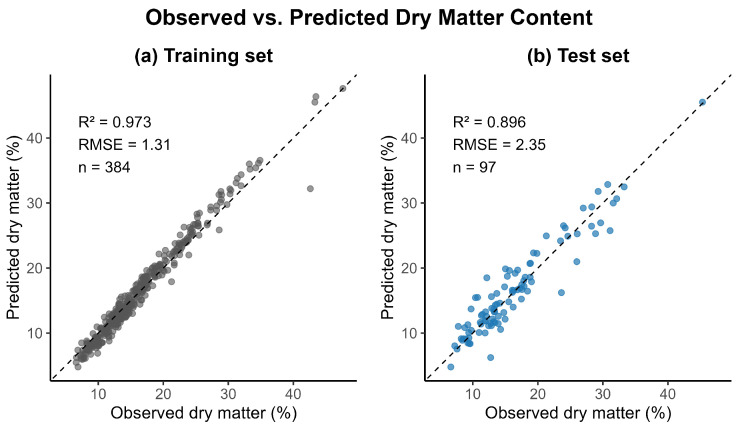
Observed versus predicted dry matter content (%) for the stacked ensemble model. Panels show (**a**) training (n=384) and (**b**) test data (n=97). The dashed line indicates perfect agreement. The ensemble achieved $R^{2}=0.973$ (RMSE = 1.31%) on training and $R^{2}=0.896$ (RMSE = 2.35%) on test data.

The resulting stacked model achieved strong predictive performance for dry matter estimation (see Figure 2). On the training dataset (n=384), the ensemble attained a coefficient of determination of $R^{2}=0.973$ with a root-mean-square error (RMSE) of 1.31 percentage points. Importantly, this performance generalized well to the independent test dataset (n=97), yielding $R^{2}=0.896$ and an RMSE of 2.35. These results indicate that the stacked ensemble improved predictive accuracy relative to individual base learners while maintaining robust generalization to unseen data.

Although overall accuracy provides a useful summary of model performance, it does not reveal how the ensemble arrives at its predictions. Additional insight can be obtained by examining the structure of the stacked model itself, in particular which base learners are retained by the meta-learner and the configurations they represent. This perspective helps to clarify how different modeling approaches contribute to the final predictions and which types of information from the hyperspectral data are most influential within the ensemble.

**Figure 3:**
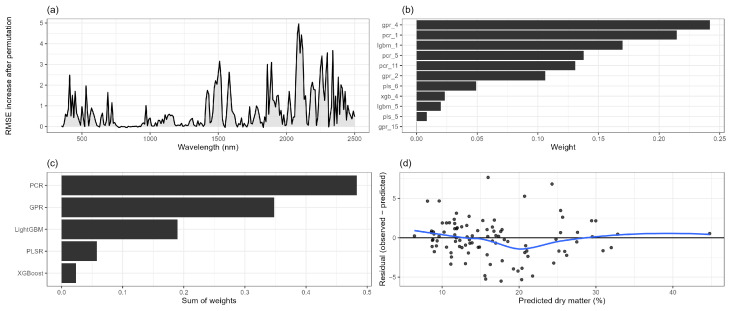
Panel (**a**) shows spectral sensitivity of the stacked model, quantified using permutation importance of contiguous 10 nm wavelength bands on the independent test set; larger values indicate a greater increase in prediction error after permutation. Panel (**b**) displays the meta-learner (non-negative LASSO) weights assigned to individual retained base learners, highlighting their relative contributions to the ensemble. Panel (**c**) summarizes the aggregate contribution of each model family, obtained by summing meta-learner weights across retained candidates within each family. Panel (**d**) presents residuals versus predicted dry matter content for the test set, where grey points represent individual observations and the blue LOESS curve indicates the smoothed trend used to assess potential systematic prediction bias.

Table 7 summarizes the base learners retained by the meta-learner together with their corresponding hyperparameters and weights. Although only a subset of candidate models received non-zero weights, these retained learners spanned multiple model families, indicating that the ensemble benefited from structurally diverse representations of the hyperspectral signal. To further interpret how these retained models collectively behave, Figure 3 provides a diagnostic and interpretative view of the final stacked ensemble.

Figure 3 provides a detailed interpretation of the internal behavior and diagnostic properties of the final stacked ensemble model. Together, the four panels offer insight into which spectral regions drive predictive performance, how individual base learners contribute to the ensemble, and whether systematic prediction errors are present.

Panel (a) reveals a strongly structured spectral sensitivity pattern for the stacked ensemble, indicating that predictive performance is driven by distinct wavelength regions rather than being uniformly distributed across the spectrum. The largest increases in RMSE after permutation occur in the shortwave infrared (SWIR), with pronounced sensitivity peaks spanning approximately 1400–1600 nm and 1900–2300 nm. These regions coincide with major absorption features associated with leaf water content, cellulose, lignin, and overall dry matter composition, underscoring their central role in dry matter estimation. Secondary sensitivity peaks are observed in the red-edge and near-infrared region (approximately 700–900 nm), suggesting an additional contribution from the leaf internal structure and canopy scattering effects. In contrast, the visible spectrum below roughly 600 nm exhibits comparatively low sensitivity, indicating that reflectance in this range provides limited independent information for dry matter prediction once longer wavelengths are taken into account. Overall, the spectral importance profile demonstrates that the stacked model relies predominantly on SWIR wavelengths linked to biochemical and water-related absorption processes, while integrating complementary structural information from the red-edge and near-infrared regions. This pattern is consistent with established physical interpretations of hyperspectral reflectance in vegetation and supports the use of full-spectrum modeling approaches for dry matter estimation.

Panels (b) and (c) summarize the composition of the stacked ensemble in terms of meta-learner weights. Panel (b) shows the weights assigned to individual retained base learners, while panel (c) aggregates these contributions at the model-family level. The results demonstrate that the meta-learner allocates substantial weight to multiple Gaussian process regression and principal component regression candidates, alongside additional contributions from partial least squares regression and gradient-boosted tree models. Notably, several models from the same family are retained, indicating that distinct hyperparameter configurations can capture complementary aspects of the hyperspectral signal even within a single modeling paradigm. In contrast, Elastic Net regression and Random Forest models were not retained in the final ensemble, suggesting that their predictive information was largely redundant with that provided by other learners. For Elastic Net, this likely reflects the dominance of latent-variable representations in capturing linear structure, while for Random Forests, the lack of retention may be attributed to overlap with more expressive boosting-based models that better exploit nonlinear interactions in high-dimensional spectral data. Overall, the ensemble composition highlights the benefit of combining latent-variable models, which emphasize the global spectral structure, with nonlinear learners capable of modeling localized interactions.

Panel (d) presents residuals plotted against predicted dry matter values for the test set, with a LOESS (Locally Regression) smoother included to facilitate visual assessment of potential systematic trends. Residuals are centered around zero across the prediction range, with no strong evidence of heteroscedasticity or systematic bias. A slight tendency toward underestimation at higher dry matter values is visible, which is common in hyperspectral regression problems where extreme observations are relatively scarce. Nevertheless, the absence of pronounced trends or variance inflation suggests that the stacked ensemble provides a stable and well-calibrated fit on unseen data.

Taken together, Figure 3 indicates that the stacked model achieves its improved predictive performance by integrating complementary spectral information across wavelength regions and leveraging structurally diverse base learners, while maintaining robust generalization and well-behaved residuals.

## 4. Discussion

The results of this study indicate that simple narrow-band spectral indices are generally insufficient for robust prediction of leaf dry matter content across heterogeneous samples. In the present dataset, even the best-performing physiologically motivated index (NDMI) explained only a limited fraction of the variance, while water-sensitive indices yielded near-zero predictive power. This behavior is consistent with long-standing spectroscopic theory and empirical evidence showing that dry-matter-related absorption features arise from cellulose, hemicellulose, and lignin, which produce broad and overlapping absorption complexes in the SWIR region rather than isolated spectral features [52,53]. As a consequence, ratio-type indices targeting individual bands tend to saturate quickly and fail to generalize when spectral or structural variability increases. The limited performance of narrow-band water and structural indices in the present study is not inconsistent with the well-established presence of water absorption features in the near- and shortwave-infrared regions. Rather, it reflects the heterogeneous structure of the dataset. The sampled leaves differed not only in water status but also in species identity, anatomical structure, developmental stage, and growth conditions. Under such conditions, reflectance variation arises from overlapping contributions of water absorption, cellulose and lignin content, internal leaf scattering, and thickness-related structural effects. Ratio-type indices targeting isolated wavelength pairs are therefore unable to disentangle these interacting sources of variability. Previous studies reporting strong correlations between SWIR indices and dry matter content were typically conducted within relatively homogeneous species groups or controlled experimental conditions, where structural variability is constrained. As biological heterogeneity increases, the predictive stability of narrow-band indices decreases, while multivariate full-spectrum approaches become more robust.

At the same time, previous studies have shown that simple dry-matter-sensitive spectral indices can exhibit moderate to strong associations with dry matter content under constrained conditions. For example, Wang et al. (2013) [40] reported strong pairwise correlations between normalized SWIR indices (e.g., NDMI, DMCI) and dry matter-related variables in relatively homogeneous vegetation samples, particularly when water absorption effects were accounted for. However, these relationships were evaluated as within-dataset associations rather than as independently validated predictive models, and their robustness is therefore expected to decrease as biological and structural heterogeneity increases. A similar pattern has been observed for more physically based approaches. The PROSPECT model, a physically based leaf radiative transfer model that simulates leaf reflectance and transmittance spectra as a function of biochemical constituents and structural properties, provides a mechanistic framework for estimating leaf traits through model inversion. PROSPECT-based inversions and physically motivated index formulations have demonstrated good performance for dry-matter-related traits under controlled experimental conditions, particularly when prior constraints on leaf structure and water content are imposed [54]. However, these studies also report increased uncertainty and reduced transferability when models are applied across species or experimental conditions, indicating that the robustness of index-based and inversion approaches is strongly dependent on the validity of their underlying assumptions.

In contrast, multivariate models that exploit information across the full hyperspectral feature space consistently yield higher predictive accuracy than narrow-band approaches. Linear latent-variable methods such as Partial Least Squares Regression (PLSR) have demonstrated strong performance for structurally driven leaf traits when applied to full-spectrum data. For example, Serbin et al. (2019) [15] reported coefficients of determination approaching $R^{2}\approx 0.89$ for leaf mass per area across biomes and functional groups, highlighting the effectiveness of multivariate spectral representations for complex structural traits, while earlier spectroscopic work emphasized the necessity of multivariate analysis to capture broad and overlapping absorption features associated with dry matter constituents [52]. Recent studies have further shown that nonlinear machine learning models can provide additional improvements by capturing higher-order interactions and local spectral structure. For instance, Yang et al. (2020) [6] demonstrated that machine learning approaches using multivariate spectral inputs substantially reduced prediction errors for dry matter estimation relative to single-index methods. Similarly, Casas et al. (2014) [17] and Kothari et al. (2023) [16] reported consistent gains from nonlinear learners when predicting structurally driven leaf traits across species and functional groups. In line with these findings, the multivariate models evaluated here outperformed narrow-band baselines.

Importantly, no single multivariate learner consistently dominated across validation folds in the present study, indicating that different model classes captured partially distinct aspects of the spectral–trait relationship. Similar behavior has been noted in hyperspectral trait modeling studies, where model rankings vary across datasets and trait types, especially when structural and biochemical signals overlap [6,16]. This complementarity motivated the use of stacked ensemble learning in the present work.

Although the stacked model achieved very high performance on the training data (*R*^2^ = 0.973), the reduction to *R*^2^ = 0.896 on the independent test set reflects expected generalization behavior in high-dimensional regression problems. Ensemble models can closely approximate training data due to their ability to combine complementary base learners; however, some reduction in performance on unseen data is consistent with the standard bias–variance trade-off. In the present study, several mechanisms mitigate excessive overfitting, including prior dimensionality reduction, regularization within individual base learners, and constrained weight estimation at the stacking stage. The observed performance gap is therefore moderate and indicative of normal generalization dynamics rather than severe overfitting.

The absolute performance gain from stacking over the strongest individual model was modest, but this is consistent with ensemble learning theory. When base learners are already well tuned and individually strong, stacked ensembles typically provide incremental improvements by reducing model-specific bias and variance rather than dramatically increasing peak accuracy [18,49]. In the present study, the primary benefit of stacking was improved consistency across validation folds and better integration of complementary linear and nonlinear representations of the spectral signal, rather than a large increase in headline performance metrics.

Taken together, these results reinforce a conceptual distinction between traits dominated by sharp, spectrally localized absorption features and those characterized by distributed biochemical structure. While water content can often be approximated using targeted indices with high accuracy, dry matter content emerges as a spectrally diffuse trait that generally requires multivariate modeling of the full reflectance signal. Within this context, stacked learning provides a rigorous framework for integrating complementary linear and nonlinear representations of hyperspectral information, yielding more robust predictions without reliance on a single modeling assumption.

Reliable, non-destructive assessment of leaf dry matter content is important for crop monitoring and precision agriculture applications. The present results demonstrate that full-spectrum hyperspectral data combined with machine learning can provide accurate estimation of this trait across heterogeneous plant material. Nevertheless, practical deployment remains challenging because Vis–NIR reflectance is influenced by multiple interacting factors, including pigment concentrations, biochemical composition, and leaf structural characteristics. For operational use, future research should focus on identifying spectral regions most strongly and consistently associated with dry matter content across species, developmental stages, and environmental conditions. Clarifying the underlying spectral response mechanisms would support the design of simplified and cost-effective sensing systems, thereby facilitating high-throughput trait monitoring and improved crop management strategies.

## 5. Conclusions

1. Leaf dry matter content cannot be reliably predicted using narrow-band spectral indices or hand-crafted SWIR features, with the best set explaining only 39% of variance ($R^{2}=0.386$).
2. Combining multiple indices using Elastic Net regression improves performance ($R^{2}=0.69$) but remains insufficient for accurate dry matter estimation.
3. Full-spectrum machine learning models capture substantially more information, with a stacked ensemble of PLSR, GPR, and gradient boosting achieving $R^{2}=0.896$ on the test set.
4. Ensemble performance arises from combining latent linear structures with nonlinear refinements, highlighting the complementary strengths of different algorithms.
5. The trait cannot be reduced to a single wavelength, and integrating information across the hyperspectral signal provides both methodological and mechanistic advantages.
6. The approach is suitable for high-throughput trait estimation and can be extended to other traits or environmental conditions. Future work will focus on improving model transferability and incorporating uncertainty quantification for operational plant monitoring.
7. Transfer of the modeling approach to data acquired with different spectrometers may require recalibration and retraining using representative samples, as differences in spectral resolution, wavelength alignment, and noise characteristics can affect predictions. However, the stacked ensemble framework and preprocessing strategy remain applicable, and dimensionality reduction helps capture spectral structure that is robust across measurement systems.

## Figures and Tables

**Table 1:** Growing conditions, cultivation setup, and sampling stages of experimental plants.

Plant	Variety	Year	Growing Environment	Growth Setup	Developmental Stage at Sampling (BBCH)	Variable	Replicates
Maize (*Zea mays*)	Gucio	2024	VP	VC	30–32; 36–39; 50–59 60–69; 70–79	WS; WN	6
Faba bean (*Vicia faba*)	Bartek	2024	VP	VC	31–33; 35–39; 50–59 60–69; 70–79	WS; WN	6
Leaf radish (*Raphanus sativus*)	Sango	2024	GH	VC	30–39	FM; FO	4
Basil (*Ocimum basilicum*)	Tuscany	2024	GH	VC	50–59; 60–69	FM; FO; FG	6
Cucumber (*Cucumis sativus*)	Berlioz	2024	GH	VC	70–79	FM; FO; FG	5
Common beans (*Phaseolus vulgaris*)	Landrace	2025	GH	VC	70–79	FM; FO	5
Leaf mustard (*Brassica juncea*)	Red Giant	2025	GH	VC	12–14; 15–19; 31–37	L1; L2; L3	5
Lettuce (*Lactuca sativa*)	Rosaine	2025	GC	VC	12–14; 15–19; 31–38	L1; L2; L3	5
Aragula (*Eruca sativa*)	Coltivata	2025	GC	VC	12–14; 15–19; 31–39	L1; L2; L3	5
Potatoes (*Solanum tuberosum*)	Jogla; Monta; Prelma	2025	VP	VC	20–29; 31–39; 51–59	F0; F1; F2	3
Tomatoes (*Solanum lycopersicum*)	Betalux	2025	GH	VC	22–29; 51–59; 61–69 71–72; 73–75	W1; W2	6
Tomatoes (*Solanum lycopersicum*)	Betalux	2025	GH	VC	22–29; 51–59; 61–69 71–72; 73–75	M0; M1	6
Peas (*Pisum sativum*)	Ostinato	2025	EF	FE	61–65	S0; S1	4
Winter wheat (*Triticum aestivum*)	Zeppelin	2025	EF	FE	61–65	S0; S1	4

Abbreviations: VP, GH, GC, EF—growing environments; VC, FE—growth setups; WS, WN, FM, FO, FG, L1–L3, F0–F2, W1–W2, M0–M1, S0–S1—experimental variables.

**Table 2:** Descriptive statistics of leaf dry matter content (%) in the training and testing subsets.

Subset	*n*	M	Me	SD	Min	Max	Skew	Kurt.
Training sample	384	16.11	14.26	6.55	6.61	47.61	1.45	5.91
Testing sample	97	16.46	14.28	7.22	6.62	45.31	1.29	4.71

M—mean; Me—median; SD—standard deviation; Min—minimum; Max—maximum; Skew—skewness; Kurt.—kurtosis.

**Table 3:** Spectral indices and SWIR features evaluated as predictors of leaf dry matter content.

Index	Formula	Reference
Normalized Difference 820/1600 (NDII)	$\frac{R_{820}-R_{1600}}{R_{820}+R_{1600}}$	https://www.indexdatabase.de (accessed on 10 August 2025)
Water Index (WI)	$\frac{R_{900}}{R_{970}}$	Penuelas et al., 1997 [27]
Simple Ratio Water Index (SRWI)	$\frac{R_{860}}{R_{1240}}$	Zarco-Tejada et al., 2003 [28]
Leaf Water Index (LWI)	$\frac{R_{1300}}{R_{1450}}$	Seelig et al., 2008 [29]
Normalized Difference Lignin Index (NDLI)	$\frac{\log\left(1/R_{1680}\right)-\log\left(1/R_{1754}\right)}{\log\left(1/R_{1680}\right)+\log\left(1/R_{1754}\right)}$	Serrano et al., 2002 [30]
Cellulose Absorption Index (CAI)	$R_{2000}-0.5\times \left(R_{2100}+R_{1900}\right)$	Nagler et al., 2003 [31]
Normalized Difference Water Index 1640 (NDWI 1640)	$\frac{R_{858}-R_{1640}}{R_{858}+R_{1640}}$	Chen et al., 2005 [32]
Normalized Difference Water Index (NDWI)	$\frac{R_{860}-R_{1240}}{R_{860}+R_{1240}}$	Gao, 1996 [33]
Normalized Difference Vegetation Index (NDVI)	$\frac{R_{760}-R_{670}}{R_{760}+R_{670}}$	https://www.indexdatabase.de (accessed on 10 August 2025)
Normalized Water Index (NWI)	$\frac{R_{970}-R_{900}}{R_{970}+R_{900}}$	Babar et al., 2006 [34]
Salinity and Water Stress Index 1 (SWSI1)	$\frac{R_{803}-R_{681}}{\sqrt{R_{905}+R_{972}}}$	Hamzeh et al., 2013 [35]
Salinity and Water Stress Index 2 (SWSI2)	$\frac{R_{803}-R_{681}}{\sqrt{R_{1326}+R_{1507}}}$	Hamzeh et al., 2013 [35]
Salinity and Water Stress Index 3 (SWSI3)	$\frac{R_{803}-R_{681}}{\sqrt{R_{972}+R_{1174}}}$	Hamzeh et al., 2013 [35]
Moisture Stress Index (MSI)	$\frac{R_{1600}}{R_{820}}$	Hunt and Rock, 1989 [36]
Normalized Difference Infrared Index (NDII 2)	$\frac{R_{819}-R_{1649}}{R_{819}+R_{1649}}$	Jackson et al., 2004 [37]
Normalized Difference 1070/1200 (NDWI-Hyperion)	$\frac{R_{1070}-R_{1200}}{R_{1070}+R_{1200}}$	Ustin et al., 2002 [38]
Diseases and Water Stress Index 1 (DWSI-1)	$\frac{R_{800}}{R_{1660}}$	Apan et al., 2004 [39]
Diseases and Water Stress Index 2 (DWSI-2)	$\frac{R_{1660}}{R_{550}}$	Apan et al., 2004 [39]
Diseases and Water Stress Index 3 (DWSI-3)	$\frac{R_{1600}}{R_{680}}$	Apan et al., 2004 [39]
Diseases and Water Stress Index 4 (DWSI-4)	$\frac{R_{550}}{R_{680}}$	Apan et al., 2004 [39]
Diseases and Water Stress Index 5 (DWSI-5)	$\frac{R_{800}-R_{550}}{R_{1660}+R_{680}}$	Apan et al., 2004 [39]
Normalized Dry Matter Index (NDMI)	$\frac{R_{1649}-R_{1720}}{R_{1649}+R_{1720}}$	Wang et al., 2011 [40]
Dry Matter Content Index (DMCI)	$\frac{R_{2305}-R_{1495}}{R_{2305}+R_{1495}}$	Romero et al., [41]

$R_{\lambda }$ denotes the reflectance at wavelength λ (nm).

**Table 4:** Overview of hyperparameter tuning strategies for base learners and the stacked ensemble. All tuning was conducted using repeated 10-fold cross-validation (3 repeats) on the training set only. Reported ranges indicate the full bounds explored in the analysis scripts.

Model	Tuned Hyperparameters	Explored Range
PCR	Number of components (*num_comp*)	10–150
PLSR	Number of components (*num_comp*)	2–120
Elastic Net	*num_comp*, penalty (λ), mixture (α)	*num_comp*: 10–150; λ: $10^{-6}$–$10^{0}$ (log scale); α: 0–1
Random Forest	*num_comp*, number of trees, *mtry*, *min_n*	*num_comp*: 20–150; trees: 500–2000; *mtry*: 5–150; *min_n*: 2–20
XGBoost	*num_comp*, trees, learning rate, depth, *min_n*, *mtry*, sampling parameters	*num_comp*: 15–150; trees: 500–2500; learning rate: $10^{-3.5}$–$10^{-1.0}$; depth: 3–8
LightGBM	*num_comp*, trees, learning rate, depth, *min_n*, *mtry*, sampling parameters	*num_comp*: 10–120; trees: 600–3000; learning rate: $10^{-3.5}$–$10^{-1.0}$; depth: 3–8
Gaussian Process Regression	*num_comp*, RBF kernel bandwidth (σ)	Two-stage search: *num_comp*: 10–120; σ: $10^{-3}$–$10^{1}$
Stacked ensemble	Meta-learner regularization (λ)	Fixed value: $\lambda =10^{-6}$ (non-negative LASSO on out-of-fold predictions)

All predictors were standardized prior to model fitting. For tree-based models, *mtry* denotes the number of predictors randomly sampled at each split, and *min_n* denotes the minimum number of observations required to form a terminal node. Except for PLSR, all learners were trained on PCA-transformed spectra, with the number of retained components (*num_comp*) treated as a tunable hyperparameter.

**Table 5:** Test set performance of narrow-band indices and SWIR-derived features used as simple predictors of leaf dry matter content. Only indices with a coefficient of determination larger than 0.001 are shown.

Index	RMSE	$R^{2}$
NDMI	5.63	0.386
DMCI	6.13	0.271
CAI	6.59	0.159
NDVI	6.68	0.135
DWSI-4	6.76	0.115
DWSI-3	6.84	0.092
SWSI-2	6.95	0.063
LWI	6.96	0.062
SWSI-3	6.96	0.060
SWSI-1	6.97	0.059
NDWI-Hyp	7.13	0.014
MSI (1600–820)	7.15	0.009
NDII	7.15	0.008
NDWI (1640)	7.15	0.008
NDII-2	7.16	0.006
DWSI-1	7.17	0.005

RMSE—root-mean-square error; $R^{2}$—coefficient of determination from the independent test set (n=97).

**Table 6:** Predictive performance of individual regression models trained on PCA or PLS reduced spectral reflectance data for leaf dry matter content. Reported values are from the independent test set (n=97). RMSE is expressed in units of dry matter percentage, and $R^{2}$ indicates explained variance or the squared Pearson correlation between observed and predicted values.

Model	RMSE	$R^{2}$
LightGBM	2.49	0.879
Linear Regression (PCR)	2.61	0.871
PLSR	2.62	0.871
Elastic Net	2.60	0.871
Random Forest	2.86	0.842
Gaussian Process	3.10	0.814
XGBoost	3.40	0.770

RMSE—root-mean-square error; $R^{2}$—coefficient of determination.

**Table 7:** Retained base learners and their hyperparameters in the candidate-level stacking ensemble. Out of the candidate model library (top 20 configurations per model family), the LASSO meta-learner retained 11 base learners with non-zero weights.

Family	Model	Weight	*K*	σ	Trees	Depth	LR	mtry	*min_n*
GPR	gpr_4	0.242	25	0.0183	–	–	–	–	–
PCR	pcr_1	0.215	83	–	–	–	–	–	–
LGBM	lgbm_1	0.170	28	–	2983	4	0.0058	13	4
PCR	pcr_5	0.138	119	–	–	–	–	–	–
PCR	pcr_11	0.131	131	–	–	–	–	–	–
GPR	gpr_2	0.106	26	0.0183	–	–	–	–	–
PLSR	pls_6	0.049	39	–	–	–	–	–	–
XGB	xgb_4	0.023	89	–	659	7	0.0153	48	4
LGBM	lgbm_5	0.020	60	–	1866	5	0.0415	47	9
PLSR	pls_5	0.008	8	–	–	–	–	–	–
GPR	gpr_15	0.0001	28	0.0183	–	–	–	–	–

*K*—number of latent components (principal components for PCR and tree-based models, latent variables for PLSR); σ—RBF kernel bandwidth for Gaussian process regression; Trees—number of trees; Depth—maximum tree depth; LR—learning rate; mtry—number of predictors sampled at each split; *min_n*—minimum node size.

## Data Availability

The data presented in this study are available from the corresponding author upon request.

## Abbreviations

The following abbreviations are used in this manuscript:

RF	Random Forest
BBCH	A two-digit numerical coding system describing plant growth stages from germination to senescence
VIS	Visible spectral region
NIR	Near-infrared spectral region
PCR	Principal Component Regression
PLSR	Partial Least Squares Regression
GPR	Gaussian Process Regression
XGBoost	Extreme Gradient Boosting
LightGBM	Light Gradient Boosting Machine
Enet	Elastic Net Regularization
